# Exopolysaccharide production in fermented milk using *Lactobacillus casei* strains AP and AG

**DOI:** 10.3934/microbiol.2022012

**Published:** 2022-04-25

**Authors:** Hafidh Shofwan Maajid, Nurliyani Nurliyani, Widodo Widodo

**Affiliations:** Faculty of Animal Science, Universitas Gadjah Mada, Yogyakarta, Indonesia

**Keywords:** *Lactobacillus casei* AP and AG, milk fermentation, exopolysaccharides, viscosity, glycosyltransferase

## Abstract

This study evaluated the ability of two strains of bacterial starter cultures, *Lactobacillus casei* AP (AP) and *Lactobacillus casei* AG (AG), to produce exopolysaccharides (EPSs). First, the physicochemical properties of the fermented milk produced by AP and AG were assessed, including physical qualities like viscosity and syneresis and chemical qualities, such as pH, acidity, protein, lactose, fat content, and total solid. Then, AP and AG's ability to produce EPS was measured. Additionally, the EPS' microstructure was observed using a scanning electron microscope, and its chemical structure was assessed using Fourier transform-infrared (FT-IR) spectroscopy. Also, AP and AG's ability to produce EPS was tracked at the molecular level by studying the glycosyltransferase (*gtf*) gene. Statistical analysis showed that the milk fermented using AP and AG had similar physicochemical qualities (P > 0.05) but significantly different physical qualities (P < 0.05). Additionally, the milk fermented with AP had lower viscosity (1137.33 ± 34.31 centiPoise) than AG (1221.50 ± 20.66 centiPoise). In addition, the milk fermented using AP had higher syneresis (19.42%) than AG (17.83%). The higher viscosity and lower syneresis in the milk fermented using AG were associated with AG's ability to produce more EPS (1409 mg/L) than AP (1204 mg/L). In addition, according to the FT-IR analysis, the AP- and AG-synthesized EPS contained absorption bands at 3323, 2980, 2901, 1642, 1084, 1043, and 873 cm^−1^. The absorption band at 1642 and 2980 cm^−1^ corresponds to carbonyl and methylene groups, respectively. Absorption band 873 cm^−1^ is characteristic of the α-glycosidic bond of α-glucan in EPS. Moreover, the absorption bands on the wavelength region corresponding to the functional groups in the AP- and AG-produced EPS were similar to those in commercially available EPS. Lastly, *gtf*, contributing to EPS synthesis, was found in the genomes of AP and AG, suggesting the role of glycosyltransferase in the EPS synthesis by both strains.

## 1. Introduction

Exopolysaccharides (EPSs) are extracellular biological polymers secreted from various types of microbes, including bacteria, in response to extreme environmental stress. EPS is biodegradable, non-toxic, biocompatible, and widely available in nature [1]. In addition, EPS is broadly used in the pharmaceutical and food industries because they offer many health benefits and improve the physicochemical properties of food products [2],[3].

Based on their structures, EPSs are divided into two types, homopolysaccharides (HoPSs) and heteropolysaccharides (HePSs) [4]. A HoPS is composed of only one type of monosaccharides, mostly D-glucose or D-fructose. A HoPS composed of glucose is linked via *α*-glycosidic or by β-glycosidic bonds, producing α-glucans or β-glucans. Meanwhile, a HoPS consisting of fructose is generally linked via β-glycosidic bonds to form levans and inulin [3]. In contrast, a HePS consists of 2 to 8 monosaccharides, mainly D-glucose, D-galactose and L-rhamnose. In addition, acetylated monosaccharides, including N-acetyl-glucosamine (GlcNAc) and N-acetyl-galactosamine (GalNAc), and D-glucuronic acid, D-mannose, and glycerol, are components of HePS [5].

EPS-synthesizing enzymes in bacteria are encoded by several members of the *gtf* gene family [6]. Some of these genes form an *eps* gene cluster which involved in 4 special functions, namely gene regulation for EPS synthesis, chain length determination, monosaccharide (repeating unit) synthesis, and polymerization before EPS secretion from the cells. For example, *L. casei* shirota has 14 genes that play a role in EPS synthesis in the *eps* gene cluster [7]. In addition, *epsA* and *epsE* have a crucial role in EPS synthesis; their expression is responsible for a cell's ability to adapt to harsh environments. For example, *epsA* plays an important role in regulating EPS biosynthesis [8]; deleting *epsA* from the EPS gene cluster in *Lactobacillus johnsonii* FI9785 results in the loss of the ability of cells to produce EPS [9].

Lactic acid bacteria (LAB) can produce EPSs [10]. The EPSs produced by LAB have been widely used because they are generally considered safe [11] and applicable for food, cosmetic and pharmaceutical industries as bioflocculants, bioabsorbents, and drug delivery agents, respectively [2]. The most prominent EPS-producing LAB genera are *Lactobacillus*, *Lactococcus*, *Leuconostoc*, *Pediococcus*, *Streptococcus, Enterococcus*, and *Weissella* [10]. In addition, some *Lactobacillus* species produce EPS and provide health benefits. LAB species that produce EPS include *Lactobacillus fermentum* [12], *Lactobacillus plantarum* [13],[14], *Lactobacillus paraplantarum* [15], *Lactobacillus rhamnosus* [16], *Lactobacillus kefiranofaciens* [17], *Lactobacillus casei* [18], and *Lactobacillus helveticus* [19].

The EPS from *Lactobacillus* can also act as an antioxidant and immunomodulator, suppresses cancer cell development, and lower cholesterol levels [12],[14],[20],[21]. Additionally, the EPS extracted from *Lactobacillus* sp. Ca6 has a skin-healing effect on male Wistar rats [22]. Moreover, the EPS produced by *Bifidobacterium* sp. reduces cholesterol levels in obese rats [23]. Additionally, the use of probiotics from the genus *Lactobacillus* as cholesterol-lowering agents is associated with the bacteria's ability to produce EPS; the probiotics' anti-hypercholesterolemic effect is attributed to the use of high EPS-producing strains. Lastly, the addition of cholesterol in the fermentation medium can stimulate EPS synthesis in *L. delbrueckii* [24].

According to Widodo *et al*. [25], the *Lactobacillus casei* strain AP and AG isolated from the feces of Indonesian infants can function as probiotics. In addition, both *L. casei* AP and AG strains can inhibit the growth of *Bacillus cereus* and *Escherichia coli*; they can attach to the mucus cells *in vitro* and are highly viable when grown at pH 2.0 and 1.5% bile salt [25]. Also, the milk fermented using *L. casei* AG has a higher viscosity than the milk fermented using *L. casei* AP. The differences in viscosity may be affected by several factors, including the quantity and quality of the EPS produced. Therefore, this study aimed to evaluate the ability of the *L. casei* strains AP and AG to synthesize EPS, characterize the EPS produced by them, and uncover the genes responsible for EPS synthesis.

## 2. Materials and methods

### 2.1. Bacterial Strains and culture condition

The bacterial cultures used in this study were *L. casei* strains AP and AG (The Faculty of Animal Science, Universitas Gadjah Mada) [25],[26]. The bacterial cells were sub-cultured by plating on de Man-Rogosa-Sharpe Agar (MRSA; Merck, Jakarta, Indonesia) and incubated at 37 °C for 24 h under micro-aerobic conditions.

### 2.2. Starter culture preparation and milk fermentation

*L. casei* AP and AG cells were cultured in MRS broth at 37 °C for 24 h. First, the bacterial culture at 5% (v/v) was inoculated into 18% sterilized skim milk (w/v), and the mixture was incubated at 37 °C for 10 h. Then, the bacterial cultures at this growth stage were used as starters for milk fermentation. Next, milk fermentation was carried out according to Tamime and Robinson [27]. In summary, skim milk powder (Mirota KSM, Yogyakarta, Indonesia) was added to fresh milk to achieve 18% total solid (w/v), and the mixture was pasteurized at 80 °C for 20 min. After cooling, the mixture was inoculated with 1% (v/v) bacterial cultures of *L. casei* AP and AG and incubated at 37 °C for 10 h. Lastly, the fermented milk products were stored at 4 °C until analysis.

### 2.3. Physicochemical analysis of fermented products

PH values were determined using a pH meter (PT-70, Boeco, Germany). Protein concentrations were measured using the Lowry method [28]. Milk fat content (%) was quantified using the Gerber method; meanwhile, lactose content was determined using the titration method [29]. Total solids were measured according to Baird and Bridgewater [30]. Viscosity was quantified using a viscometer, and the values were expressed in centipoise (cP). Syneresis was determined using the centrifugation method (Eppendorf 5804 R: Merck, Jakarta, Indonesia) according to Keogh dan O'Kennedy [31]. Lastly, all data were presented as the mean ± standard deviation of three measurements of each sample.

### 2.4. Exopolysaccharide isolation and purification

Exopolysaccharide (EPS) isolation was conducted based on the method in Kanamarlapudi and Muddada [32] with a modification. First, *L. casei* strains AP and AG were grown in 500 mL of MRS broth supplemented with 50 g/L sucrose (Sigma Aldrich, Jakarta, Indonesia) and incubated at 37 °C for 24 h. Then, the supernatant was separated from the biomass via centrifugation at 4000 × g at 4 °C for 30 min. Next, the supernatant was precipitated by mixing with 3 volumes of cold 95% ethanol and incubating at 4 °C for 48 h. Afterward, the precipitated EPS was separated by centrifugation at 11000 × g for 20 min. Then, the pellet was diluted with 12% trichloroacetic acid (TCA; Sigma Aldrich, Jakarta, Indonesia) and incubated at 4 °C for 24 h. Afterward, the dilution was centrifuged at 11000 × g at 4 °C for 60 min, and the harvested EPS was dissolved in double-distilled water. Finally, the EPS solution was dialyzed using Centrikon (Pall, Nanosep centrifugal devices) at 4 °C for 48 h to remove small molecules or ions residues.

### 2.5. Microstructure analysis of EPS

The microstructure of EPS was analyzed using scanning electron microscopy (SEM) based on the method by Allain *et al*. [33]. First, the harvested EPS was fixed onto glass coverslips with 15 µL of 2.5% glutaraldehyde at 4 °C for 2 h. Then, the coverslips were washed with 0.1 M phosphate buffer for 30 min, and samples were dehydrated using an ethanol gradient of 20, 50, 70, 80 and 100% (w/v). After drying with acetone, the coverslips were coated with 30 mM platinum using an auto fine coater for 90 s. Then, the samples were examined using SEM (JSM-5700; Jeol, Tokyo, Japan). SEM images were taken at magnifications between 1,000 to 5,000 ×.

### 2.6. Fourier Transform Infrared (FTIR) analysis of EPS

The structure of EPS was elucidated by analyzing the different functional groups using Fourier transform-infrared (FTIR) spectroscopy based on the method by Kanamarlapudi and Muddada [32]. First, 3 mm compressed disks were prepared by mixing 2 mg of lyophilized EPS with 200 mg of KBr. Next, the spectrum was corrected for the KBr background. Then, the pellets were scanned in the range of 4000 to 500 cm^−1^ with resolution of 4 cm^−1^ and for 32 scans.

### 2.7. Amplification of the glycosyltransferase (gtf) gene and phylogenetic tree construction

The amplification of *gtf* was performed using PCR with specific primers [34] in a 25 µL reaction mixture. The mixture consisted of 12.5 µL of Master Mix (GoTaq^®^, Green Master Mix, USA), 1 µL of forward primer, 1 µL of reverse primer, 1 µL of template DNA, and 9.5 µL of nuclease-free water. PCR cycling conditions included preliminary denaturation at 95 °C for 5 min, followed by 35 cycles of denaturation at 95 °C for 30 s, amplification at 58 °C for 45 s, and elongation at 72 °C for 45 s, and final elongation at 72 °C for 5 min. DNA visualization was done using 1.5% agarose gels with a 1000-bp DNA ladder as the marker. The amplified bands were sequenced, and the resulting nucleotide sequences were aligned with the nucleotide database using the Basic Local Alignment Search Tool (https://blast.ncbi.nlm.nih.gov/Blast.cgi). The phylogenic tree was built using MEGA 7 with a maximum likelihood algorithm.

### 2.8. Statistical analysis

The data were expressed as mean ± standard error of the mean (SEM). Statistical analysis was conducted using SPSS Version 16.0 (Statistical Product and Service Solutions, IBM Corporation, New York, USA). Statistical comparisons were carried out using paired t-tests with the significance set at a P-value less than 0.05; differences with P-values less than 0.01 were considered highly significant.

## 3. Results and discussion

### 3.1. Physicochemical qualities of fermented milk

The physicochemical qualities of the milk fermented using *L. casei* AP and AG strains were measured (Table 1). First, the physical qualities were measured based on the syneresis (in %) and viscosity (in centipoise; cP) of the products. Meanwhile, the chemical qualities of the products were measured based on their protein, fat, and lactose content, pH, acidity, and total solid (%).

**Table 1.** Physiochemical qualities of the milk fermented using *L. casei* AP and AG.

Parameters	*L. casei* AP	*L. casei* AG
pH ^ns^	4.54 ± 0.01	4.45 ± 0.01
Acidity^ns^ (%)	0.80 ± 0.01	0.92 ± 0.01
Protein^ns^ (%)	8.62 ± 0.92	8.34 ± 0.83
Fat^ns^ (%)	3.56 ± 0.01	3.41 ± 0.05
Lactose^ns^ (%)	4.36 ± 0.03	5.46 ± 0.44
Total solid ^ns^ (%)	16.40 ± 0.36	16.12 ± 0.53

*Note: ^ns^: not significant.

The chemical qualities of the milk fermented using *L. casei* strains AP and AG were not different (P > 0.05) (Table 1), indicating that *L. casei* strains AP and AG produced similar fermented milk products. Furthermore, *L. casei* strains AP and AG were isolated from the same source, i.e., the feces of Indonesian infants less than 1 month old who had only been fed breast milk [25], suggesting that both strains had similar nutrient profiles.

Milk fermentation using LAB as starter cultures produces lactic acid, thus increasing the acidity and decreasing the pH of the culture [27],[29]. Therefore, an increase in acidity and a decrease in pH indicate that the fermentation processes are working properly. The pH of milk fermented using *L. casei* AP and AG were similar (P > 0.05) at 4.54 and 4.45, respectively (Table 1). Accordingly, *L. casei* AP and AG strains produced fermented products with similar acidity at 0.80 ± 0.01 and 0.92 ± 0.01%, respectively, consistent with the normal acidity level of fermented milk at 0.7 to 0.9% (v/v), according to Walstra *et al*. [35]. In addition, these data suggest that both *L. casei* strains AP and AG are effective in acidifying milk, as pH 4.5 is the isoelectric point that triggers casein coagulation in milk, forming a curd and increasing the viscosity of a product. The acidity of fermented milk affects its qualities, such as texture, aroma, and taste.

The milk fermented using *L. casei* strains AP and AG had similar protein, fat, lactose, and total solid content (P > 0.05) (Table 1). The protein content in the fermented milk products using *L. casei* strain AP and AG was 8.62 and 8.34%, respectively, higher than the minimum protein content requirement of fermented milk at 2.7% by Codex Alimentarus [36]. The protein content of the fermented milk products was also higher than those in a previous study by Widodo *et al*. [37], who reported the protein content was 4.66% in the milk fermented using *L. casei* AP. The addition of skim milk to the fresh milk before fermentation likely contributed to the high protein content in the fermented products. On the other hand, the fermented milk products using *L. casei* AP and AG had comparable fat content at 3.56 and 3.41%, respectively (P > 0.05), within the maximum requirement of fat content at 10% [36]. Also, the lactose content in the milk fermented using *L. casei* AP and AG, at 4.36 and 5.46%, respectively, did not differ significantly (P > 0.05) (Table 1). These data indicated that both bacterial strains had similar enzymatic capabilities of using lactose as an energy during fermentation. A similarity in total solid content was also observed in the fermented milk products using *L. casei* AP and AG, at 16.12 and 16.40%, respectively (P > 0.05). The total solid in a fermented milk affects its syneresis as casein, and other solids in milk increase the water-binding capacity, thus decreasing the syneresis.

**Table 2.** Physical quality of the milk fermented using *L. casei* AP and AG.

Parameters	*L. casei* AP	*L. casei* AG
Syneresis (%)	19.42 ± 0.66 ^a^	17.83 ± 0.77 ^b^
Viscosity (cP)	1137.33 ± 34.31 ^a^	1221.50 ± 20.66 ^b^

*Note: ^a,b^ P < 0.05 in the same line.

The physical qualities of the milk fermented using *L. casei* AP and AG were compared (Table 2). Syneresis is a separation of fluid that occurs during milk fermentation; high syneresis is not expected in fermented milk products. The syneresis of the milk fermented using *L. casei* AP was higher than that using *L. casei* AG (P < 0.05). The syneresis rate was 19.42% for the milk fermented using *L. casei* AP and 17.83% for the milk fermented using *L. casei* AG. Syneresis is closely related to whey formation in cheese production. Higher whey production leads to higher syneresis. On the other hand, viscosity, a parameter for determining milk flow, is influenced by the composition and concentration of milk components, pH, and temperature. For example, casein and milk fat are the main components contributing to milk viscosity [38]. Here, the viscosity of the milk fermented using *L. casei* AG was higher than that using *L. casei* AP (P < 0.05) (Table 2). The viscosity of the milk fermented using *L. casei* AG was 1221.50 ± 20.66 cP, whereas that of the milk fermented using *L. casei* AP was 1137.33 ± 34.31 cP. The viscosity of fermented milk is strongly influenced by the formation of curd during fermentation. In addition, viscosity is affected by the production of high-molecular-weight EPS. EPS is widely used in the food industry due to its ability to improve the physicochemical properties of food products [2]. EPS, widely used as a stabilizer, thickener, emulsifier, and gelling agent, has a good water-binding capability. In addition, EPS interacts with milk protein to increase viscosity [39].

### 3.2. EPS production

Exopolysaccharides (EPSs) are extracellular polysaccharides with high molecular weights [40]. EPSs are composed of monosaccharides and non-carbohydrate substituents, such as acetate, phosphate, pyruvate, and succinate. According to Lee *et al*. [41], EPS can be produced in two forms, capsular polysaccharide, which is tightly attached to the surface of bacterial cells such as forming a capsule, and slime polysaccharide (SPS), which is loosely attached or even secreted from the cell.

**Figure 1.**
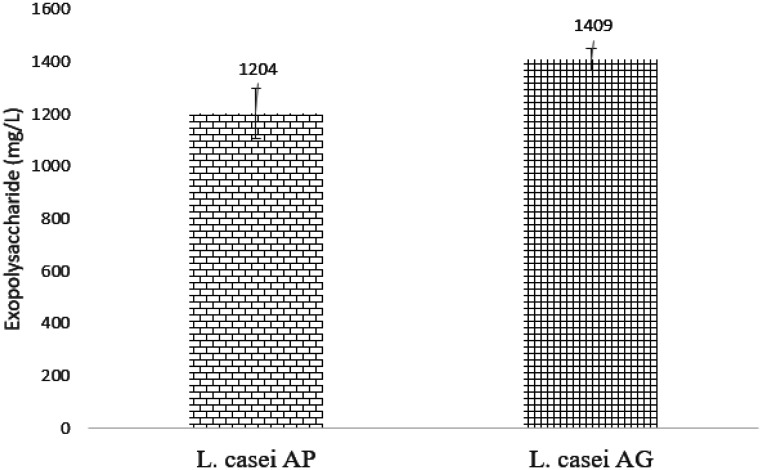
EPS production (mg/L) by *Lactobacillus casei* AP and AG.

The production of EPS in the milk fermented using *L. casei* AP and AG were compared (Figure 1). EPS production in the milk fermented using *L. casei* AG was higher at 1409 mg/L than that using *L. casei* AP at 1204 mg/L (P < 0.05), in agreement with the previous findings by Kanamarlapudi and Muddada [32] and Rajoka *et al*. [16]. Kanamarlapudi and Muddada [32] reported an EPS production at 1950 mg/L by *Streptococcus thermophilus* when grown in skim milk at 30 °C. Meanwhile, Rajoka *et al*. [16] reported an EPS production at 461–737 mg/L by 6 *Lactobacillus rhamnosus* strains. In addition, another study [42] reported EPS productions in the range of 50–500 mg/L by other LAB. The difference in the ability to biosynthesize EPS between *L. casei* AP and AG suggests that the ability to produce EPS is strain-dependent. The higher EPS production in the milk product using *L*. *casei* AG is correlated with the higher viscosity of the milk product.

### 3.3. Microstructure of EPSs

**Figure 2.**
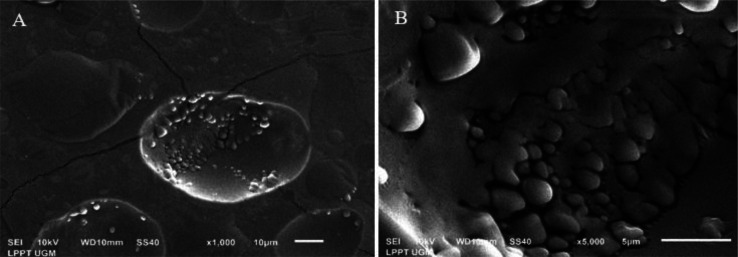
Visualization of the EPSs produced by *L. casei* AP under scanning electron microscopy. (A) EPSs at 1000 × magnification. (B) EPSs at 5000 × magnification.

**Figure 3.**
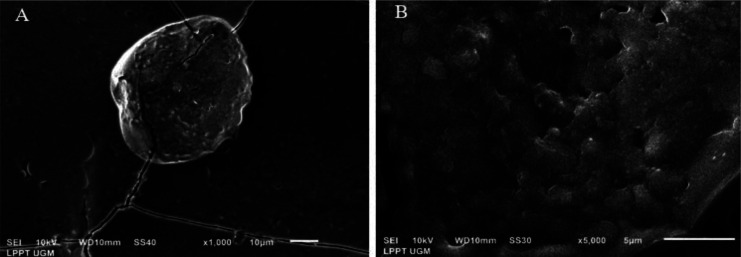
Visualization of the EPSs produced by *L. casei* AG under scanning electron microscopy. (A) EPSs at 1000 × magnification. (B) EPSs at 5000 × magnification.

The small particles of EPSs were examined using SEM. The EPSs produced by *L. casei* AP (Figure 2) and AG (Figure 3) were visualized at 1000 and 5000 × magnification.

EPS production occurs during the stationary growth phase to respond to the stress caused by nutrient depletion [43]. In this study, *L. casei* AP and AG cells were grown to stationary phase before EPSs were harvested. SEM analysis showed that the EPSs produced by *L. casei* AP and AG were irregular lumps with a coarse surface (Figures 2 and 3).

### 3.4. Chemical structure of EPS

**Figure 4.**
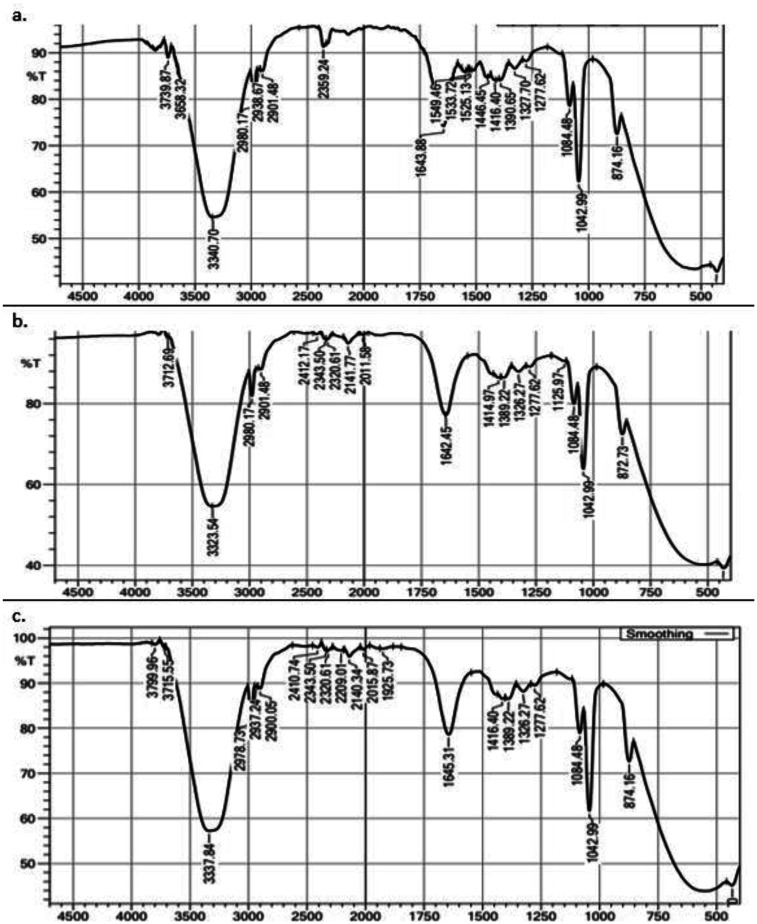
FTIR Spectra of EPS. (a) The EPS standard. (b) The EPS produced by *L. casei* AP. (c) The EPS produced by *L. casei* AG.

Fourier transform-infrared (FT-IR) spectroscopy is an analytical method based on the different vibrational frequencies of various chemical bonds. In this study, FT-IR spectroscopy detected the functional groups in the EPS standard (Figure 4a) and the EPS produced by *L. casei* AP (Figure 4b) and *L. casei* AG (Figure 4c); in addition, their covalent bonds were characterized. The FT-IR spectra contained absorption bands at 3323, 2980, 2901, 1642, 1084, 1043, and 873 cm^−1^ (Figure 4). A broad stretching at 1658 cm^−1^ represents the stretching vibration of carboxyl groups, while the absorption band 2938 cm^−1^ corresponds to C-H stretching of methyl or methylene groups usually present in hexose like glucose or galactose, or deoxyhexoses like rhamnose or fucose [44]. Absorption band 1057 cm^−1^ relates to amide groups, while the absorption band 879 cm^−1^ is characteristic of the α-glycosidic bond of α-glucan [45].

The FT-IR spectrum of EPS produced by *L. casei* AP and AG in this study was identical with that FT-IR spectra of EPS standard available commercially. This finding agreed with a previous report by Chowdhury *et al*. [46] and Shankar *et al*. [47]. Chowdhury *et al*. [46] reported FT-IR spectra of the EPS samples consisting of absorption bands at 3385, 2981, 1648, and 1423 cm^−1^, which were characteristic of -OH, C-H, C-C, and CH_3_ groups, respectively. A broad band at 1648 cm^−1^ corresponded to carboxyl groups [46]. In addition, Shankar *et al*. [47] have observed the FT-IR spectra of carboxyl and hydrogen bonds in an EPS sample. Overall, the FT-IR spectrum data in this study displayed the same pattern of group carboxyl, methyl groups, and hydrogen bound observed in other EPS samples [46],[47].

### 3.5. Amplification and sequencing of the glycosyltransferase gene

The glycosyltransferases (*gtf*) gene was amplified using specific primers, and the amplified bands were visualized using 1.5% (w/v) agarose gel electrophoresis. The amplification of *gtf* using the specific primers was confirmed to generate a 325-bp fragment (data not shown). The presence of *gtf* was associated with the ability of *L. casei* AP and AG to produce EPS (Table 1; Figures 2 and 3). According to Tieking *et al*. [48], glycosyltransferase is involved in EPS biosynthesis in *Lactobacillus*. In addition, glycosyltransferase catalyzes the formation of glycosidic bonds for the polymerization of monosaccharides [49]. HoPS synthesis involves specific pairs of glycosyltransferase and substrates, such as dextransucrase and dextran or levansucrase and levan [50].

**Figure 5.**
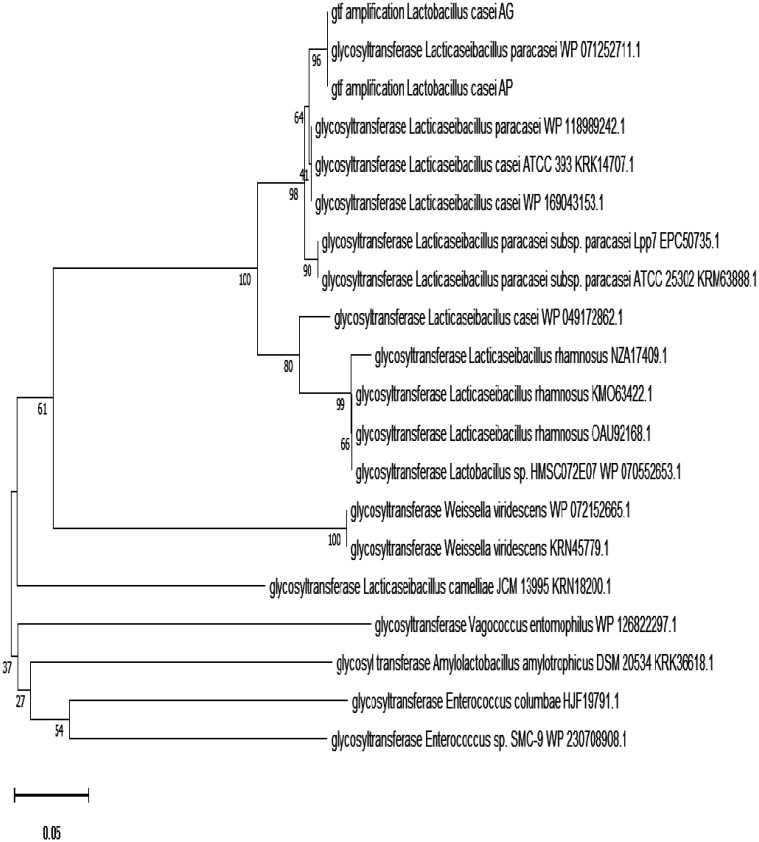
The phylogenetic tree of the gtf genes originated from L. casei AP and AG.

Sequencing analysis of the amplified *gtf* of *L. casei* AP and AG uncovered a 325-bp product. BLASTX analysis of these sequences revealed high similarity (97%) with several corresponding sequences of the genes encoding glycosyltransferases. Sequence homology and accession number from several glycosyltransferase-encoding genes were applied as a database to construct the phylogenetic tree (Figure 5). The phylogenetic tree clearly showed that the *gtf* gene amplified from the genomic DNA of *L. casei* AP and AG were clustered within a group of glycosyltransferase genes from *Lacticaseibacillus paracasei*.

The biosynthesis of heteropolysaccharides (HePSs) is a complex process due to the involvement of more than one glycosyltransferase and housekeeping enzymes. First, housekeeping enzymes synthesize nucleotide sugars, such as UDP-glucose, dTDP-glucose, UDP-galactose, and dTDP-rhamnose. Then, these nucleotide sugars are converted to repeating units of monosaccharides using several specific glycosyltransferases [51],[52]. Next, the repeating units of monosaccharides are assembled on a C55-isoprenoid-lipid carrier molecule attached to the cytoplasmic membrane [6]. Finally, the synthesized sugar chains are secreted from the cell as EPSs.

The glycosyltransferase-encoding gene (*epsE*) is crucial in the biosynthesis of long-chain EPSs. *epsE* encodes the priming glycosyltransferase, also known as undecaprenyl-phosphate glycosyl-1-phosphate transferase, that plays a role in the early stages of the preparation of EPS. The undecaprenyl-phosphate glycosyl-1-phosphate transferase carries the first sugar-1-phosphate (monosaccharide) to a lipid carrier molecule bound to the cell membrane [34]. Mutations in the *epsE* eliminate the ability of *L. lactis* NIZO and *L. rhamnosus* to produce EPS [53],[54]. In addition, Leeber *et al*. [53] showed that mutations in *epsE* in *Lactobacillus rhamnosus* GG decreased the number of EPS produced by up to 3 times compared to the wild type and affected the synthesis of long-chain EPS composed of galactose.

### 4. Conclusions

This study finds that a higher viscosity and a lower syneresis in milk products fermented using *L. casei* AG are associated with higher exopolysaccharide production. In addition, the ability to produce EPS is strongly associated with the glycosyltransferase (*gtf*) genes.
